# Sherlock: A Semi-automatic Framework for Quiz Generation Using a Hybrid Semantic Similarity Measure

**DOI:** 10.1007/s12559-015-9347-7

**Published:** 2015-08-04

**Authors:** Chenghua Lin, Dong Liu, Wei Pang, Zhe Wang

**Affiliations:** Department of Computing Science, University of Aberdeen, Aberdeen, AB24 3UE UK; BBC Future Media and Technology - Knowledge and Learning, BBC Bridge House, MediaCityUK, Salford, M50 2QH UK; College of Computer Science and Technology, Jilin University, Changchun, 130012 China

**Keywords:** Quiz generation, Linked data, RDF, Educational games, Semantic similarity, Text analytics

## Abstract

In this paper, we present a semi-automatic system (Sherlock) for quiz generation using linked data and textual descriptions of RDF resources. Sherlock is distinguished from existing quiz generation systems in its generic framework for domain-independent quiz generation as well as in the ability of controlling the difficulty level of the generated quizzes. Difficulty scaling is non-trivial, and it is fundamentally related to cognitive science. We approach the problem with a new angle by perceiving the level of knowledge difficulty as a similarity measure problem and propose a novel hybrid semantic similarity measure using linked data. Extensive experiments show that the proposed semantic similarity measure outperforms four strong baselines with more than 47 % gain in clustering accuracy. In addition, we discovered in the human quiz test that the model accuracy indeed shows a strong correlation with the pairwise quiz similarity.

## Introduction

Big Data analytics is one of the areas of fast-growing importance as it provides ways in which one can make sense and effective use of data. Among the Big Data landscape, one important territory is linked data which rise from the Semantic Web community [14]. By interlinking heterogeneous data sources in a standardised format, linked data are highly structured and machine-readable and thus are suitable for the tasks involving knowledge representation and management such as interactive games [5] and question answering [30], to name a few. In particular, interactive games have been proven to be an effective way for facilitating knowledge exchange between humans and machines and have attracted great research interest intersecting the fields of computing science and cognitive science [3, 16].

On the one hand, efforts have been made to design games with the purpose of semi-automating a wide range of knowledge transfer tasks by leveraging the wisdom of the crowd. For instance, symmetric and asymmetric verification games have been developed for assisting Semantic Web tasks such as ontology building, ontology alignment, content annotation and entity interlinking [13, 26]. Likewise, quiz-like games have also been developed to rank, rate and clean up linked data [31, 32]. In this way, factual knowledge is transferred from humans, especially domain experts, to computers.

On the other hand, work has also been done to unleash the potential of linked data in generating educational quizzes for aiding learners’ knowledge acquisition from a knowledge base [1, 5]. When building a quiz generation system using linked data, existing approaches [1, 5] are based on domain-specific templates and the creation of quiz templates relies on ontologists and linked data experts, preventing end-users from participating in quiz authoring. Without user participation, such systems potentially limit the diversity and variation of quizzes that the system may otherwise offer. Rey et al. [25] moved one step forward regarding the domain-dependent issue by introducing a quiz generation mechanism that is applicable to different linked data repositories. Nevertheless, their system still lacks a generic linked data-enabled framework for semi-automatically creating quizzes related to different topics.

Moreover, a system that can generate quizzes with different difficulty levels will better serve users’ needs. From a cognitive science perspective, Aponte et al. [2] argued that the difficulty of challenges greatly influences the aesthetics of a game and thus plays a central role in game design. However, such an important feature is rarely offered by existing systems. Waitelonis et al. [31] determined the difficulty of a quiz by simply assessing the popularity of an RDF resource, without considering the fact that the difficulty level of a quiz is directly affected by the selection of wrong candidate answers. Also, the most common way of generating wrong candidate answers is to randomly select them from the results of querying linked data repositories and hence provides no means to control the difficulty level during the process of quiz generation. Furthermore, while different similarity measures have been widely used for measuring the degree of closeness or separation of target objects, the problem of how well similarity measures can be used to represent the degree of knowledge difficulty in terms of human perception still remains relatively unexplored.

In this paper, we propose a novel semi-automatic quiz generation system (Sherlock) empowered by semantic and machine learning technologies [20, 21]. Sherlock is distinguished from existing quiz generation systems in a few aspects: (1) a mechanism based on a novel hybrid semantic similarity measure is introduced for controlling the difficulty level of the generated quizzes; (2) Sherlock offers a generic framework for generating quizzes of multiple domains with minimum human effort; and (3) it provides a user-friendly interface allowing users to easily create customised quizzes.

In order to control the difficulty level of the generated quizzes, a novel linked data (LD)-based hybrid semantic similarity measure, called TF-IDF (LD), is proposed. To investigate how well the proposed algorithm can be used to represent difficulty levels (i.e. *difficult*, *medium* and *easy*) of knowledge, we evaluated the proposed TF-IDF (LD) algorithm on the BBC Wildlife dataset.^1^ We compare the performance of TF-IDF (LD) against four strong baselines, i.e. two knowledge-based and two text-based similarity measures. It was observed that the knowledge-based measures gave better performance when predicting the *easy* class compared with the text-based measures, but they are inferior in the prediction for the *difficult* and *medium* classes. Our proposed hybrid semantic measure TF-IDF (LD) outperforms four strong baselines (see section “Experimental Results”) and gives at least 50 % gain in clustering accuracy for all the three classes. Furthermore, Sherlock also provides a generic framework for generating quizzes of multiple domains with minimum human effort, and its effectiveness has been evaluated on datasets from three different domains.

The rest of the paper is organised as follows. We first review the related work in section “Related Work”, followed by the presentation of the Sherlock architecture in section “The Sherlock Architecture”. The hybrid semantic similarity algorithm is detailed in section “The Linked Data-Based TF-IDF Algorithm”. Experimental results are reported and discussed in section “Experiment”, and we finally conclude the paper in section “Conclusion and Future Work”.

## Related Work

### Games with a Purpose and Educational Games

A series of symmetric and asymmetric verification games was presented in [26] with the aim to motivate humans to contribute to building the Semantic Web. *BetterRelations* [13] is a representative symmetric verification game built following the concepts of “games with a purpose”, which attempts to solve the problem of ranking RDF triples within the description of an entity. Other quiz-like games [31, 32] focus on ranking, rating and cleansing linked data. The assumption underlying these games is that the frequency of a question being correctly answered implies the importance of the supporting linked data used to create the quiz. However, the focus of these games is to harness human intelligence to perform tasks that cannot be automated, rather than creating learning experiences for humans.

In contrast to games with a purpose, Damljanovic et al. [5] presented a template-based method for generating educational quizzes. In addition, a conversational AI agent was introduced to guide the learners and dynamically select quizzes according to the learners’ needs. Linked Data Movie Quiz (LDMQ)^2^ is another representative work of using linked data for template-based quiz generation [25]. LDMQ is able to generate quizzes related to a user-selected actor or actress, asking questions about the director, the release date or the characters of a film in which the actor or actress has appeared. The question and correct answers are directly derived from the results of SPARQL^3^ queries against the Linked Movie Data Base (LMDB) [12], whereas the incorrect answers are randomly chosen from a set of candidates collected following some handcrafted rules.

One of the common limitations shared by existing quiz generation systems is the domain-dependent issue. That is when applying the template-based quiz generation method to a new domain, significant human efforts must be required on tasks such as creating new question templates, writing SPARQL queries according to a domain-specific ontology and defining rules for collecting wrong answers for a quiz. Again, these tasks are not trivial for non-domain experts such as teachers, content editors and mainstream web users.

In addition, most of the existing quiz generation systems endeavour to automate the quiz creation task to the largest extent without providing the functionality for manual quiz creation. However, allowing manual question authoring from end-users is important because it can increase both the level of user engagement and topic diversity of the generated quizzes. Moreover, creating quizzes offers the creator the opportunity of *teaching someone else*, which is the lowest level of the Learning Pyramid.^4^ It is also arguably true that quiz players tend to retain more knowledge during the process of creating their own quizzes.

Finally, quizzes with varying difficulty levels are important for formal learning. However, as stated in [31], many quiz generation systems have the same limitation that the generated quizzes being either “too simple or too difficult”, largely due to the lack of quantitative analysis on the relationship between the wrong candidate answers and the correct one(s). This has in turn motivated us to develop a systematic way of measuring quiz difficulty level using semantic similarity measures.

### Similarity Measures

A similarity (distance) measure reflects the degree of closeness or separation of the target objects, and it must be determined before performing clustering. In this work, we tackle the research challenge of how to predict the difficulty levels of quizzes perceived by humans in terms of similarity measures, which to our knowledge, has not been studied in previous work. Therefore, we review some of the most representative similarity measures in the literature, which serve as the ground for our preliminary experiments.

#### Corpus-Based Approaches

Measures of text similarity have been used for a long time in natural language processing applications and related areas. Corpus-based measures aim to identify the degree of similarity between text units using statistical patterns of words derived from large corpora, where the most representative measures are cosine similarity, averaged Kullback–Leibler divergence (KLD) and the squared Euclidean distance [15].

Cosine similarity is one of the most popular similarity measures and has been widely used in information retrieval and text clustering applications [15]. When text documents are represented as term vectors, the similarity of two documents corresponds to the inner product space of the two vectors, i.e. the cosine of the angle between them. The averaged Kullback–Leibler divergence (KLD), rooted from information theory-based clustering, evaluates the differences between two probability distributions. By modelling a document as a probability distribution over terms, the similarity of two documents is then transformed as the distance between two corresponding probability distributions. Some more advanced approaches rely on word co-occurrence patterns derived from large corpus, which indicate the degree of statistical dependence between text units. Such statistical dependences can then be used for measuring text similarity. Representative approaches along this line include pointwise mutual information (PMI) [29] and latent semantic analysis (LSA) [18].

#### Knowledge-Based Approaches

In contrast to corpus-based approaches that are purely oriented on statistical techniques, knowledge-based approaches rely on human-organised knowledge (e.g. Semantic Network, WordNet and Linked Open Data) to encode relations between a collection of concepts [7, 9, 23].

WordNet [7] is a large English lexical knowledge database in which terms are grouped into different sets known as synsets with a list of synonyms. A number of measures have been developed based on the WordNet hierarchy such as accessing the semantic relatedness of words/entities [33] and identifying word sense under different contexts [19]. Wu and Palmer [33] proposed to measure the semantic similarity of two concepts by considering the depth of these two concepts in the WordNet taxonomy as well as the depth of the least common subsumer (LCS). Similarly, Resnik [24] measured semantic similarity between words by counting the shared edges between two concepts in the taxonomy.

The closest work to our proposed hybrid semantic similarity measure is the linked data semantic distance (LDSD) [23], which also uses the graph information in RDF resources or semantic similarity measure and has been adopted by a music recommendation system [22]. The similarity computation results of LDSD purely rely on statistics on the direct and indirect in and out connections among RDF resources of DBpedia. Working on top of DBpedia gives LDSD the possibility of covering many various domains. However, when comparing to our proposed hybrid semantic similarity measure, apart from providing no means to weigh the importance of different predicates, LDSD also cannot deal with literal values and textual descriptions in a RDF dataset.

## The Sherlock Architecture

**Fig. 1:**
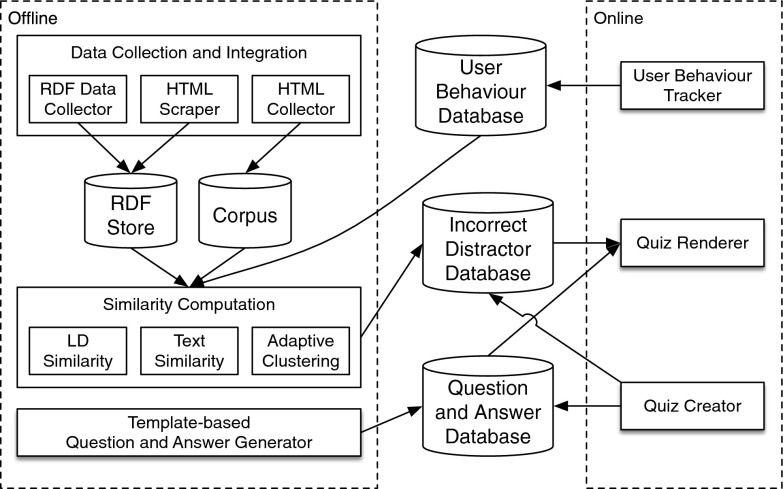
Overall architecture of Sherlock

In this section, we present the details of the architecture of our proposed Sherlock framework. Figure 1 depicts an overview of the framework, in which the components are logically divided into two groups: online and offline. Within the Sherlock framework, different components can interact with each other via three shared databases that respectively containing information about: (1) user behaviours, (2) questions and answers of quizzes and (3) distractors (i.e. incorrect answers). The live Sherlock system can be accessed from http://sentinet-mango.abdn.ac.uk/.

### Data Collection and Integration

We collected two different types of data: (1) structured RDF data published by DBpedia and the BBC and (2) unstructured text describing objects (entities) collected from the BBC website and Wikipedia. These datasets play two main roles, namely serving as the knowledge base for quiz generation and calculating the similarity scores between objects (entities). Detailed descriptions on dataset preparation are given in section “Data”.

### Similarity Computation

The similarity computation module is the core of the offline part of Sherlock. The similarity computation module first accesses the RDF store and the text corpus, and it then calculates the similarity scores between each object/entity pair. In the second step, the module performs *K*-means clustering to partition the wrong candidate answers into different difficulty levels according to their similarity scores with respect to the correct answer of a quiz. Here, we empirically set $K=3$, which corresponds to three predefined difficulty levels, i.e. “easy”, “medium” and “difficult”.

### Template-Based Question and Answer Generator

The quiz generator component adopts a template-based method similar to Linked Data Movie Quiz (LDMQ), which is able to boost up the system in the situation of cold start and/or coping with data from a new domain. For instance, a template “Which of the following animals is {?animal_name}?” can be instantiated by replacing the variable with rdfs:label of an animal.

### Quiz Renderer

**Fig. 2:**
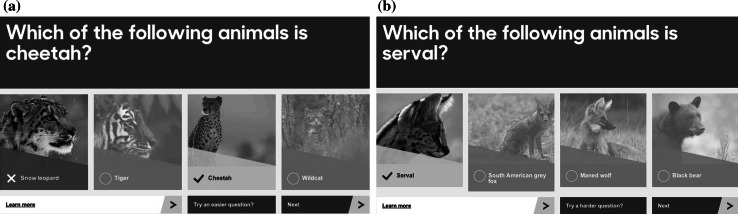
User interface for playing quizzes. **a** User interface when an incorrect choice is made. **b** User interface when a correct choice is made

The quiz renderer module realises the user interface through which users can interact with the system, as shown in Fig. 2. The question and correct answer are retrieved from a dedicated database, whereas the wrong answer candidates are selected from the results calculated by the similarity computation module. It is worth noting that the foaf:depiction attribute in the RDF store provides links to the images used to render the quizzes.

To encourage the users to carry on their learning journeys, the “learn more” link on the bottom left of the interface points to a web page containing information about the correct answer as shown in Fig. 2. In addition, the system gives users changes to tune down (or up) the difficulty level of the next quiz, depending on whether a user fails a difficult quiz or succeeds in an easy one.

### Quiz Creator

We believe it is necessary to allow users to create their own questions and answers in order to make the game more attractive, engaging as well as making the topic of the quizzes more diverse. The quiz creator module allows users to create customised quizzes with their own questions and images. For instance, one can take a picture of several ingredients and let people guess what dish one is going to cook. More detailed discussion about the quiz creator module is given in section “Customised Quiz Authoring”.

### User Behaviour Tracker

When a user is playing a quiz, the user behaviour tracker keeps records of the identification of the user, the ID and correct answer of the quiz, and the user-selected answer.

## The Linked Data-Based TF-IDF Algorithm

In this section, we describe the main algorithm we have developed in Sherlock. As we recall, one of the key challenges in our work is to measure the difficulty levels of quizzes. To this end, we developed a hybrid similarity measure by combining a novel linked data-based TF-IDF scheme with the classical text-based cosine similarity measure, called TF-IDF (LD).

Typically, RDF datasets are formalised as graphs, and the direct and indirect distances in those graphs can be used to measure the similarity between RDF resources, as in the case of linked data semantic distance (LDSD) [23]. While LDSD is reported to be effective on large-scale datasets such as DBpedia and Freebase, the importance of predicates in RDF resources is not considered, which limits the accuracy of LDSD. To address this issue, we propose a novel linked data-based TF-IDF scheme by mapping Named Graphs into vectors, which takes the predicate information into account. The resulting linked data-based TF-IDF vectors are then combined with the cosine similarity measure to calculate the semantic similarity between two RDF resources. Before describing the proposed algorithm, we first give formal definitions to the following technical terms: *term*, *sentence*, *document* and *corpus*.

### **Definition 1**

*A sentence and a term*

An RDF statement, i.e. a tuple of *(subject, predicate, object)*, is defined as a *sentence*. A combination in the form of *(subject, predicate)* or *(predicate, object)* is regarded as a *term*.

For example, (_:Cheetah, wo:family, _:Felidae) is a sentence, whereas (_:Cheetah, wo:family) and (wo:family, _:Felidae) are two terms in the sentence. Here, wo is the namespace of BBC Wildlife Ontology.^5^

### **Definition 2**

*A document and a corpus*

A Named Graph that is related to an RDF resource, e.g. an animal, a recipe or a painting, is a *document*, which may contain multiple RDF statements. A collection of RDF documents is a *corpus*.

For example, the RDF statements shown in Listing 1 constitute a document. This document contains three sentences describing the animal cheetah.
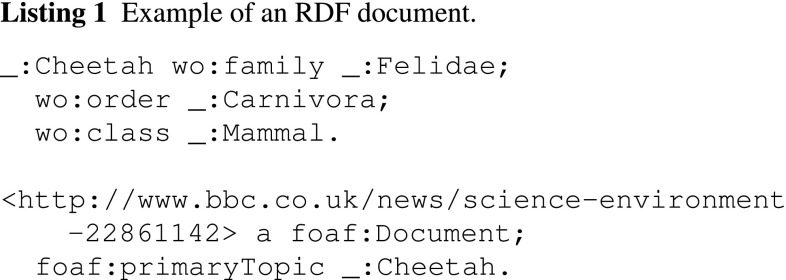


### **Definition 3**

*The relation between a term, a sentence and a document*

If a document $d$ contains a sentence $(s, p, o)$, we then say terms $(s, p)$ and $(p, o)$ are *in* document $d$, i.e. $(s, p) \in d$ and $(p, o) \in d$.

### The TF-IDF (LD) Algorithm

We now describe our TF-IDF (LD) algorithm. With the definitions above, the classical TF-IDF scheme can then be applied to the RDF datasets. That is given a term *t*, a document $d$ and a corpus *C*, the *Term Frequency (TF)* and the *Inverse Document Ffrequency (IDF)* are calculated as follows:

1 $$\begin{aligned} tf(t, d)&= {} \left\{ \begin{array}{ll} 1 &{}  \text {if}\,t \in d\\ 0 &{}  \text {if}\,t \notin d \end{array} \right. \end{aligned}$$

2 $$ idf(t, C)= \log \frac{| C |}{| \{d \in C: t \in d\} |} $$

In information retrieval (IR), a standard *Term Frequency (TF)* function calculates the number of times a term has appeared in a text document. In contrast, the *Term Frequency* function of our proposed TF-IDF (LD) algorithm, as shown in Eq. (1), is a Boolean function as there is no term co-occurrences in an RDF document graph. This means if a term [e.g. (*s*, *p*) or (*p*, *o*)] has appeared in an RDF document, its term frequency is 1, and 0 otherwise. Equation (2) calculates the *Inverse Document Frequency (IDF)*, where the numerator is the total number of RDF documents in corpus *C* and the denominator is the total number of RDF documents in *C* that contain term *t*. By applying Eqs. (1) and (2) to RDF documents *a* and *b*, they can be transformed to linked data-based TF-IDF vectors (e.g. ${\mathbf {t}}_a$ and ${\mathbf {t}}_b$), based on which we can then calculate the semantic similarity between these two RDF documents using cosine similarity as shown in Eq. (3).

3 $$ \text {SIM}_C ({\mathbf {t}}_a, {\mathbf {t}}_b) = \frac{{\mathbf {t}}_a \cdot {\mathbf {t}}_b}{\Vert {\mathbf {t}}_a \Vert \Vert {\mathbf {t}}_b \Vert }. $$

A summary of the TF-IDF (LD) algorithm is given in Algorithm 1.
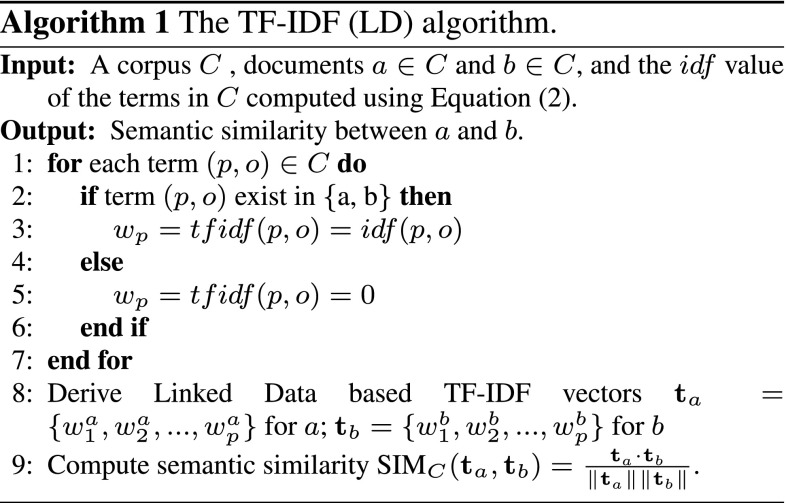


## Experiment

In this section, we first explore how well similarity measures can be used to suggest quiz difficulty levels that match human perception. In another set of experiments, we further evaluate Sherlock as a generic framework for quiz generation by testing the system on datasets from three different domains. In particular, we aim to investigate the following two research questions:

1. Can similarity measure(s) be used as appropriate means for measuring quiz difficulty levels?
2. To what extend can the quiz difficulty level suggested by similarity measure(s) match human perception on knowledge difficulty level?

### A Pilot Evaluation of Quiz Difficulty Level

We shall not try to give a general definition of difficulty covering a wide range of psychological aspects from emotional problems to intellectual and physical challenges. Instead, we consider the notion of difficulty in the sense used in quiz generation, the one that is built as combinations of predefined candidates. Of course the study of the overall difficulty for a given quiz involves multiple factors such as the intellectual level of knowledge covered in the quiz and users’ knowledge background. In the preliminary study, we address the problem in a less complicated scenario, in which the difficulty level of a quiz is directly driven by the semantic similarity between the correct answer and the wrong answers.

#### Data

We conducted the preliminary experiment for measuring quiz difficulty level based on the BBC Wildlife dataset. The choice of dataset for evaluation is based on the fact that (1) there is no readily available gold standard for benchmarking from the literature; (2) in the Wildlife dataset, each animal has been labelled under the biological classification system (i.e. family, order and class), which can be naturally used as the gold standard for evaluation; and (3) according to the statistics from the BBC, the BBC Wildlife website is one of the most frequently visited BBC websites, indicating a broad public interest in the Wildlife data. In particular, we have prepared two different versions of the BBC Wildlife^6^ dataset, i.e. one based on the structured RDF data and the other based on the unstructured textual data.

##### *RDF Data*

As for the Wildlife dataset, DBpedia and the BBC Wildlife website have already published RDF data,^7^ so we harvested the structural data directly from these two data sources. In total, there are 49,897 RDF triples in the dataset.

##### *Textual Data*

In addition to the RDF data, we have also prepared a dataset by collecting textual descriptions for each entity (i.e. different animals) in the Wildlife dataset from the corresponding BBC and Wikipedia web page. Here, the textual datasets are mainly used for calculating the text-based similarity scores between entities for controlling the quiz difficulty levels. In the preprocessing, an HTML parser is used to extract contents from the HTML pages by discarding tags, contents from the navigation bar and advertisements. In the second step, we further remove wildcards, word tokens with non-alphanumeric characters and lower-case all word tokens in the dataset, followed by stop word removal and Porter stemming.^8^ The statistics of textual dataset are summarised in Table 1.

**Table 1:** Statistics of the Wildlife textual dataset

Dataset	# of docs	Avg. doc length^†^	Avg. doc length*	Vocab. size^†^	Vocab. size*
Wildlife	437	1190	652	26,004	18,237

$^\dag$ Denotes before preprocessing and * denotes after preprocessing

#### Experimental Results

To tackle the first research question, in the pilot evaluation, we formulate the problem of perceiving the difficulty level of knowledge as a similarity measure problem. The hypothesis is that if some objects (entities) share a lot of (semantically) similar properties, they tend to have higher degree of semantic relatedness with subtle difference, and hence, they are more difficult to disambiguate, and vice versa.

**Fig. 3:**
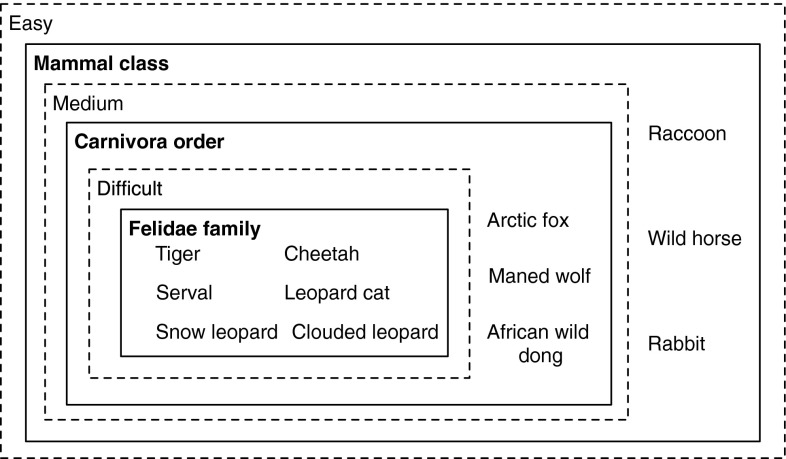
Deriving the gold standard for the BBC Wildlife dataset using the biological classification system

To derive the gold standard for the Wildlife dataset, one intuitive approach is to make use of the biological classification system. We define that if some animals have the same *family* label (e.g. Cheetah and Serval), these animals would be very similar to each other and hence *difficult* to be disambiguated. Likewise, if some animals have the same *order* label but from different *families*, they will be less similar and correspond to a *medium* difficulty level when generating a quiz. Similarly, quizzes generated based on animals with the same *class* label but different *family* and *order* labels will be most dissimilar and correspond to the *easy* level. An illustrative example of the gold standard is shown in Fig. 3.

In the pilot evaluation, we tested the proposed TF-IDF (LD) algorithm against four strong baselines in the task of measuring quiz difficulty levels. The baselines are two knowledge-based similarity measures (i.e. LDSD [23] and a WordNet-based measure called WUP [33]) using the RDF dataset; and two text-based similarity measures (i.e. cosine similarity with traditional TF-IDF and KLD) using the textual dataset.

Table 2 shows that for the text-based similarity measure, KLD outperforms TF-IDF in predicting the *difficult* and *easy* clusters while have similar performance in predicting the *medium* cluster. The knowledge-based measures slightly outperform the text-based measure for about 3 % in overall. It was also found that compared with the text-based measures, the knowledge-based measures (i.e. LDSD and WUP) give much better performance in predicting the *easy* cluster (i.e. 30 % higher), but are inferior to the prediction of the *difficult* and *medium* clusters. The proposed hybrid semantic similarity algorithm, TF-IDF (LD), outperforms all the four strong baselines for all difficulty levels, with over 47 % improvement in terms of overall accuracy. This demonstrates the effectiveness of the proposed algorithm. One reason why the proposed TF-IDF (LD) algorithm significantly outperforms the baselines is likely due to the fact that by treating RDF graphs as documents and applying the classical TF-IDF method, the proposed algorithm can capture richer semantic information from data.

**Table 2:** Clustering accuracy of different similarity measures for measuring quiz difficulty levels

Dataset	LDSD	WUP	KLD	TF-IDF	TF-IDF (LD)
	RDF	RDF	Text	Text	RDF
Difficult	18.4	2.4	37.5	29.2	**85.7**
Medium	7.9	9.3	11.4	11.6	**66.2**
Easy	82	74.5	50.9	44.8	**99.3**
Overall	36.1	28.7	33.3	28.5	**83.7**

Unit in % and numbers in boldface denote the best result in their respective row

To better compare and illustrate the clustering performance, Table 3 lists the top ten most similar animals to Cheetah^9^ found by different similarity algorithms. In this table, animals are listed in descending order based on their similarity to Cheetah, and the ones that are not in the same *family* as Cheetah are highlighted in bold. Table 3 shows that, among the four baselines, KLD performs best with three animals in the cluster not belonging to the same family as Cheetah; in contrast, WUP is least accurate with six outliers in the cluster. The TF-IDF (LD) algorithm again gives the best performance with only one outlier, i.e. *Aardvark*, being included.

**Table 3:** Top 10 most similar animals to Cheetah found by different algorithms (inappropriate ones are highlighted in bold)

WUP	KLD	TF-IDF	LDSD	TF-IDF (LD)
Jaguar	Leopard	Leopard	Lion	Serval
Lion	Lion	**Blackbuck**	**Stoat**	Snow Leopard
Serval	Cougar	Lion	Leopard	Lion
Cougar	Tiger	Leopard Cat	Tiger	Leopard
**Meerkat**	Jaguar	Cougar	Serval	Cougar
**Aardvark**	**Spotted Hyena**	Asian Golden Cat	Cougar	Wildcat
**Coyote**	Leopard Cat	**Grant’s gazelle**	**Gray Wolf**	Jaguar
**Capybara**	Snow Leopard	**Spotted Hyena**	**Red Fox**	Tiger
**Stoat**	**Bongo (antelope)**	**Blue Wildebeest**	**Meerkat**	**Aardvark**
**Indri**	**Fossa**	Snow Leopard	**Human**	Eurasian Lynx

### Using Human Judgements to Examine Quiz Difficulty Levels

Although the previous pilot experiment shows that similarity measures, especially the proposed TF-IDF (LD) algorithm, are potentially good means for measuring quiz difficulty levels, this study is still based on a synthetic gold standard without human evaluation. Therefore, it is necessary to verify whether the difficulty levels captured by similarity measures are indeed in line with human perception, and if so how well the correlation could be.

#### Task Description of Human Evaluation

To investigate the second research question, we propose a task that creates a formal setting for assessing how human perceive knowledge difficulty levels, called the quiz game task. Basically, the task involves playing quiz games, in which the subject is presented with quizzes produced using three selected similarity measures, namely LDSD, TF-IDF and TF-IDF(LD), with five quizzes generated for each difficulty level per measure. Therefore, there are altogether 45 quizzes generated based on the Wildlife dataset using the three different similarity measures. The rationales of using a subset of the baselines are mainly based on the following two considerations: 1) those four baselines performed very similar in the pilot study and 2) more importantly, four baselines plus the proposed algorithm will involved 75 test quizzes, requiring more than 15 min for a subject to complete. It was reported by Szalma et al. [28] that human evaluation test taking more than 15 min will result in the participants being less focused and more likely to be interrupted.

The above described tasks were offered on Amazon Mechanical Turk,^10^ which has been successfully used in the past to develop gold-standard data for various tasks such as natural language processing [4, 27] and images labelling [6]. We presented each subject with jobs containing 45 quiz tasks. Each job (i.e. a quiz) was performed by 30 separate subjects.

For each job, we record the answer picked by the subject. Also, to reduce the randomness of human evaluation, the subjects are instructed to choose an additional option “I don’t know”, if one is not sure about the answer of a quiz. Such a selection will be automatically categorised as an incorrect answer.

#### Model Accuracy

To quantify the difficulty levels perceived by users in the human evaluation task, we introduced the concept of *model accuracy*, which indicates the percentage of times users have chosen the correct answer of the quizzes generated by a model. Here, the model refers to a particular similarity measure (e.g. LDSD or TF-IDF ). Let $q_k^s$ be the answer selected by the *s*th subject for the *k*th quiz; $c_k$ be the correct answer for the *k*th quiz and *S* denotes the number of subjects, and the accuracy of the *k*th quiz is calculated as follows:

4 $$\begin{aligned} A_k = \sum _{s} ( q_{k}^s= c_k) / S. \end{aligned}$$

Finally, we are interested in calculating the model accuracy $$M_m^l $$, which encodes the percentage of times users have chosen the correct answer for the test quizzes of difficulty level *l* generated by model *m*. The derivation of $$M_m^l $$ is formalised in Eq. (5)

5 $$\begin{aligned} M_{m}^{l} = \sum _{k} A_{k} / D, \end{aligned}$$

where *D* is the total number of quizzes with difficulty level *l* generated by model *m*.

#### Correlation Between Model Accuracy and Similarity Distribution

In another set of experiments, we investigated the correlation between the difficulty levels suggested by similarity measures and those perceived by human as encoded in the *model accuracy*. Our hypothesis is that if the difficulty levels suggested by similarity measures are in line with human perception, the *pairwise similarity* of the quizzes should have correlations with the *model accuracy* to certain degree. Here, the *averaged pairwise similarity* of each quiz is calculated by averaging out the similarity scores between the correct answer and distractors (i.e. incorrect answers) of that quiz.

**Fig. 4:**
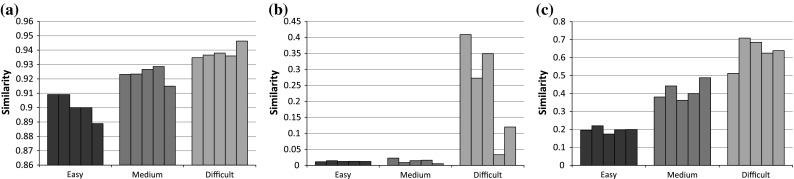
Averaged quiz similarity based on different similarity measures on the Wildlife domain dataset. **a** LDSD. **b** TF-IDF. **c** TF-IDF (LD)

In the human evaluation task, 30 subjects were presented with 45 test quizzes generated by Sherlock, i.e. 5 quizzes per difficulty level of each similarity measure (i.e. LDSD, TF-IDF and TF-IDF (LD)). Completing the whole test takes approximately 12 min for each subject on average. Next the averaged pairwise similarity of each test quiz was computed, as shown in Fig. 4.

Figure 4 shows that the pairwise quiz similarities based on LDSD are quite flat, with less than 0.06 difference between the highest and lowest value points. This is likely due to the fact that LDSD relies on the direct and indirect connections between the RDF resources, which are relatively sparse in the Wildlife RDF dataset. As a result, LDSD produces similarity values with very subtle difference. On the other hand, the classic TF-IDF scheme produces quite skewed similarity distribution, with the *easy* and *medium* classes having very small similarity values and the *difficult* class having much higher similarity scores. In contrast, the similarity distributions for each difficulty level obtained using the proposed TF-IDF (LD) algorithm are much more balanced and well spread.

**Fig. 5:**
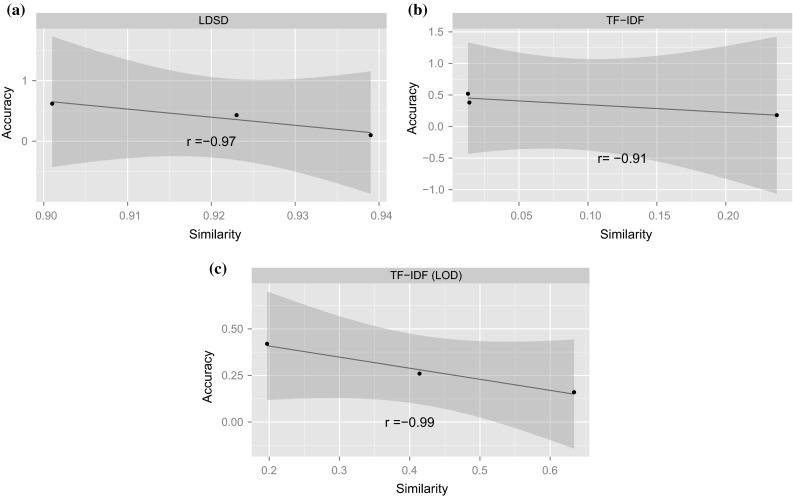
Pearson’s correlation between the model accuracy and the pairwise similarity of quizzes. **a** LDSD ($r=-0.97, p=0.156$). **b** TF-IDF ($r=-0.91, p=0.266$). **c** TF-IDF (LD) ($r=-0.99, p=0.0307$)

Figure 5 shows the Pearson’s correlation between the model accuracy and the pairwise similarity of quizzes generated from the same model, in which all the data points are the averaged value over five quizzes per difficulty level. It can be seen that for all the three tested models, model accuracy derived from human evaluation indeed shows a negative correlation with the pairwise quiz similarity. In addition, the proposed TF-IDF (LD) shows stronger correlation than both LDSD and TF-IDF in terms of the *r* value. Furthermore, for the significance test, TF-IDF (LD) is the only measure with $p \,<\, 0.05$ (cf. $p = 0.156$ for LDSD, $p = 0.266$ for TF-IDF). The human evaluation results are in line with the observations in the pilot study based on the gold standard derived from the biological classification system. Therefore, we conclude that similarity measures are good means for measuring quiz difficulty levels and that the proposed TF-IDF (LD) algorithm is superior to the baselines in the task of controlling the difficulty levels of quizzes in quiz generation.

### Domain-Independent Quiz Generation

Another key contribution of this paper is that we developed a generic framework for semi-automatic quiz generation, which can be reused in different domains with minimum human efforts. To test the framework, apart from the Wildlife domain data, we have also applied Sherlock to generate quizzes in two other domains, namely BBC Food and BBC YourPaintings.

#### Data

**Fig. 6:**
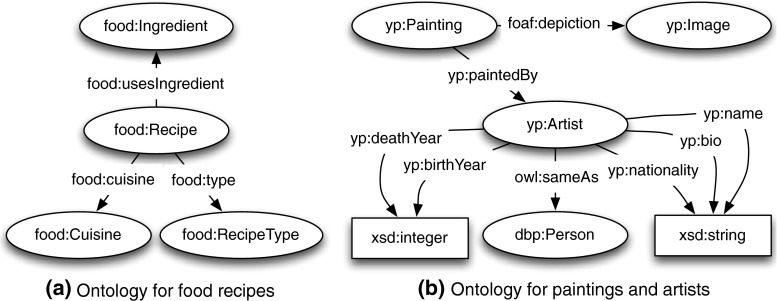
**a** Ontologies for food recipes and **b** paintings and artists. *Note* widely used predicates such as rdfs:label and rdfs:comment are omitted for making the figures more concise

Different from the Wildlife domain data, the Food^11^ and YourPaintings^12^ domains only have HTML pages available. Therefore, we first extracted information from those HTML pages and then converted it into the RDF format using two manually constructed lightweight ontologies, as shown in Fig. 6. In addition, for the purpose of incorporating the DBpedia data about painting artists into a coherent RDF store, the DBpeida Lookup API^13^ was invoked to find out the DBpedia URI for each artist, and the results were interlinked via owl:sameAs. The statistics of the RDF dataset are summarised in Table 4.

**Table 4:** Statistics of the RDF datasets from three different domain

Dataset	RDF triples	Number of species	RDF triples per recipe	Distinct objects shared by at least two recipes	Distinct subjects shared by at least two recipes
Wildlife	49,897	886	16.1	323	74
Food	55,006	5412	9.3	2419	0
YourPaintings	25,314	41	197.9	252	39

#### Quiz Generation

When generating quizzes for a new domain, the existing template-based methods [1, 5] require sophisticated rules or SPARQL queries for collecting wrong answers. In contrast, the Sherlock system, benefiting from the domain-independent similarity measures, is more flexible as there is no need to manually define rules or write SPARQL queries when applying to a new domain. To test the system, we have applied Sherlock to generate quizzes of three different domains, namely BBC Wildlife, BBC Food and BBC YourPaintings, with 321, 991 and 2315 quizzes being automatically generated for each domain, respectively.

#### Customised Quiz Authoring

Sherlock allows users to create their own quizzes and share with others, which is an important functionality not offered by other systems. Quiz authoring can not only complement automatic quiz generation for generating quizzes with more diverse topics, but also allow collaborative learning, i.e. users teach each other and learn together. We have collaborated with the editorial team in BBC Knowledge and Learning division to investigate whether it is appropriate for creating quizzes for formal learning and the outcome turned out to be very positive.

**Fig. 7:**
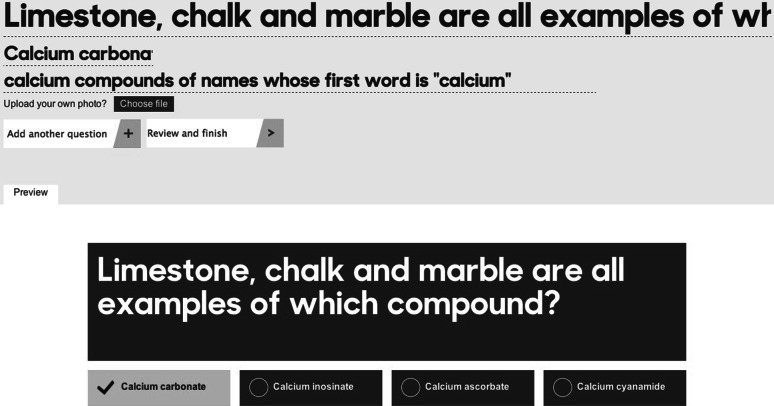
User interface for creating a quiz

Figure 7 depicts the quiz creator module.^14^ Quiz authoring involves three simple steps: (1) write a question; (2) set the correct answer (distractors are suggested by the Sherlock system automatically); and (3) preview and submit. Another advantage of the Sherlock system is that the created quizzes will not be presented exactly the same every time when they are being played, because the candidate answers are dynamically retrieved from the similarity computation component.

## Conclusion and Future Work

One of the key challenges in analysing and making effective use of Big Data is to deal with the unstructured text and natural language. Linked data, as an essential part of the Big Data landscape, interlinks heterogeneous data sources in a standardised structured format. These features make linked data easy to be consumed by machines and are particularly suitable for tasks related to knowledge engineering.

In this paper, we presented Sherlock, a generic framework for generating educational quizzes using linked data. Inspired by cognitive science studies [2], Sherlock also provides a mechanism for scaling the difficulty levels of the generated quizzes. Such a feature is deemed to have fundamental influences on the attractiveness of a game to users [2]. In summary, Sherlock offers two distinctive features compared to existing systems: (1) it provides a generic framework for generating quizzes of multiple domains with minimum human effort and (2) it introduces a mechanism for controlling the difficulty level of the generated quizzes based on a novel hybrid semantic similarity measure TF-IDF (LD). Extensive experiments show that the proposed TF-IDF (LD) algorithm outperforms four strong baselines with more than 50 % gain in predicting the difficulty level of quizzes, where similar observations have been observed in the human evaluation task.

As for future work, we first plan to carry out more comprehensive user testing and evaluation to further explore the relationship between quiz difficulty and semantic similarity. Second, it would be useful to extend the Sherlock system with natural language generation (NLG) capability to create more complicated quizzes. Third, we will consider deploying our system on the cloud by using privacy preserving approaches [8, 34]. Finally, apart from the K-means clustering algorithm used for clustering quizzes of different difficulty levels, we will consider using more advanced clustering [17, 35] and classification algorithms [10, 11] to perform better online learning of the quiz difficulty levels based on real-time user feedbacks.

## Footnotes

1 http://www.bbc.co.uk/nature/wildlife/.

2 http://lamboratory.com/hacks/ldmq/.

3 http://www.w3.org/TR/rdf-sparql-query/.

4 http://homepages.gold.ac.uk/polovina/learnpyramid/about.htm.

5 http://www.bbc.co.uk/ontologies/wildlife/2010-02-22.shtml.

6 http://www.bbc.co.uk/nature/wildlife/.

7 The corresponding ontology can be found at: http://www.bbc.co.uk/ontologies/wildlife. e.g. http://www.bbc.co.uk/nature/life/Cheetah.rdf returns RDF statements describing cheetah.

8 http://ldc.usb.ve/~vdaniel/porter.pm.

9 http://bbc.co.uk/nature/life/Cheetah.

10 http://www.mturk.com.

11 http://www.bbc.co.uk/food/.

12 http://www.bbc.co.uk/arts/yourpaintings/.

13 http://wiki.dbpedia.org/lookup/.

14 The quiz creator interface can be accessed from http://sentinet-mango.abdn.ac.uk/#/quiz/create.
